# Structure and cation distribution in perovskites with small cations at the A site: the case of ScCoO_3_

**DOI:** 10.1088/1468-6996/16/2/024801

**Published:** 2015-03-10

**Authors:** Wei Yi, Igor A Presniakov, Alexey V Sobolev, Yana S Glazkova, Yoshitaka Matsushita, Masahiko Tanaka, Kosuke Kosuda, Yoshihiro Tsujimoto, Kazunari Yamaura, Alexei A Belik

**Affiliations:** 1 International Center for Materials Nanoarchitectonics (WPI-MANA), National Institute for Materials Science (NIMS), 1-1 Namiki, Tsukuba, Ibaraki 305-0044, Japan; 2 Institute of Physics and Beijing National Laboratory for Condensed Matter Physics, Chinese Academy of Sciences, Beijing 100190, Republic of China; 3 Department of Chemistry, Lomonosov Moscow State University, Leninskie Gory, 119992 Moscow, Russia; 4 Synchrotron X-ray Station at SPring-8, NIMS, Kohto 1-1-1, Sayo-cho, Hyogo 679-5148, Japan; 5 Materials Analysis Station, NIMS, 1-2-1 Sengen, Tsukuba, Ibaraki 305-0047, Japan; 6 Materials Processing Unit, NIMS, 1-2-1 Sengen, Tsukuba, Ibaraki 305-0047, Japan; 7 Superconducting Properties Unit, NIMS, 1-1 Namiki, Tsukuba, Ibaraki 305-0044, Japan

**Keywords:** perovskites, cobaltites, high-pressure, low-spin Co^3+^ ions

## Abstract

We synthesize ScCoO_3_ perovskite and its solid solutions, ScCo_1−*x*_Fe_*x*_O_3_ and ScCo_1−*x*_Cr_*x*_O_3_, under high pressure (6 GPa) and high temperature (1570 K) conditions. We find noticeable shifts from the stoichiometric compositions, expressed as (Sc_1−*x*_*M*_*x*_)*M*O_3_ with *x* = 0.05–0.11 and *M* = Co, (Co, Fe) and (Co, Cr). The crystal structure of (Sc_0.95_Co_0.05_)CoO_3_ is refined using synchrotron x-ray powder diffraction data: space group *Pnma* (No. 62), *Z* = 4 and lattice parameters *a* = 5.26766(1) Å, *b* = 7.14027(2) Å and *c* = 4.92231(1) Å. (Sc_0.95_Co_0.05_)CoO_3_ crystallizes in the GdFeO_3_-type structure similar to other members of the perovskite cobaltite family, *A*CoO_3_ (*A*^3+^ = Y and Pr-Lu). There is evidence that (Sc_0.95_Co_0.05_)CoO_3_ has non-magnetic low-spin Co^3+^ ions at the *B* site and paramagnetic high-spin Co^3+^ ions at the *A* site. In the iron-doped samples (Sc_1−*x*_*M*_*x*_)*M*O_3_ with *M* = (Co, Fe), Fe^3+^ ions have a strong preference to occupy the *A* site of such perovskites at small doping levels.

## 1. Introduction

*AB*O_3_ perovskite-type compounds, where *A*^3+^ = Y and La-Lu and *B*^3+^ = V, Cr, Mn, Fe, Co, Ni and Ni_0.5_Mn_0.5_, and their solid solutions have been attracting a lot of attention for decades from the viewpoints of fundamental physics and practical applications [1–3]. For example, some *A*CrO_3_ compounds exhibit spin-reorientation transitions [4], and doped *A*CrO_3_ are good oxygen-ion conductors and show sensitivity toward methanol, ethanol, some gases and humidity [5]. *A*CoO_3_ compounds have been investigated a lot because of spin-state transitions in Co^3+^ ions and metal-insulator transitions [6]; *A*CoO_3_ also exhibit thermoelectric [7] and catalytic properties [8]. The large *A* site of *AB*O_3_ perovskites is usually occupied by larger cations (such as, rare earths), and the small *B* site by smaller cations (such as, transition metals). The *A*/*B* inter-site mixing is very rare in simple perovskites. In complex perovskites, the *A*/*B* inter-site and intra-site mixing can occur, and the cation distribution could significantly modify properties of materials, such as, magnetic and dielectric properties. For example, the appearance of Mg^2+^ at the *A* site in BaMg_1/3_Ta_2/3_O_3_ [9] and Mn^2+^ at the *A* site in SrTiO_3_ [10] results in increased dielectric loss, and the degree of Ni^2+^ and Mn^4+^ ordering at the *B* site in *A*Ni_0.5_Mn_0.5_O_3_ changes magnetism of the system [11].

In recent years, *AB*O_3_ perovskites have been extensively expanded to smaller *A* cations, such as, Mn^2+^, Sc^3+^, and In^3+^ [12] with expectations to find new magneto-structural coupling behaviours because of large structural distortions. Unusual physical properties were indeed found, for example, in MnVO_3_ (incommensurate magnetic ordering and metallic conductivity) [13], In_2_NiMnO_6_ (spin-induced ferroelectricity) [12] and ScVO_3_ (distinct magnetic, orbital and structural properties from other members of the *A*VO_3_ (*A*^3+^ = Y and La-Lu) family [14]). Because the difference in the sizes of the *A* and *B* cations decreases the probability of the *A*/*B* inter-site mixing is increased in such perovskites. Noticeable cation mixing or, more precisely, shifts in the composition were found in (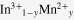)MnO_3_ (1/9 ≤ *y* ≤ 1/3) [15] and (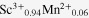)Mn_0.65_Ni_0.35_O_3_ [16]; in such perovskites, small divalent transition metals are located at the *A* site.

In this work, we investigated ScCoO_3_ perovskite and its solid solutions ScCo_1−*x*_Fe_*x*_O_3_ and ScCo_1−*x*_Cr_*x*_O_3_. We found noticeable shifts in the composition of such perovskites from ScCoO_3_ to (Sc_1−*x*_Co_*x*_)CoO_3_ and the appearance of significant amounts of small trivalent cations (Co^3+^ and Fe^3+^) at the *A* site. To the best of our knowledge, the presence of Fe^3+^ at the *A* site was detected for the first time in *AB*O_3_ perovskites.

## 2. Experimental details

Samples were prepared from stoichiometric mixtures of Sc_2_O_3_ (99.9%), Co_3_O_4_ (99.9%), Cr_2_O_3_ (99.9%), Fe_2_O_3_ (99.999%) and KClO_4_ (as the source of oxygen). The mixtures were prepared in a glove box, placed in Au capsules (in the amount of about 0.5 g for each sample) and treated at 6 GPa in a belt-type high-pressure apparatus at 1570 K for 2 h (heating rate to the desired temperature was 10 min). After the heat treatment, the samples were quenched to room temperature (RT), and the pressure was slowly released. The samples were washed in water to remove KCl obtained after the decomposition of KClO_4_.

X-ray powder diffraction (XRPD) data were collected at RT on a RIGAKU Ultima III diffractometer using CuK*α* radiation (2*θ* range of 10–100°, a step width of 0.02°, and a counting time of 2s/step). Synchrotron XRPD data were measured at 293 K on a large Debye-Scherrer camera at the BL15XU beam line of SPring-8 [17]. The intensity data were collected between 1° and 61.5° at 0.003° intervals in 2*θ*; the incident beam was monochromatized at *λ* = 0.65298 Å. The samples were packed into Lindenmann glass capillaries (inner diameter: 0.1 mm), which were rotated during measurements. Absorption coefficients were also measured, and Rietveld analysis was performed using the RIETAN-2000 program [18].

Electron probe microanalysis (EPMA) was performed using a JEOL JXA-8500F instrument. The surface of the pellets was polished on a fine alumina (0.3 *μ*m) coated film before the EPMA measurements; and Sc_2_O_3_ and Co_3_O_4_ were used as standard samples for Sc and Co, respectively.

DC magnetic susceptibilities (*χ* = **M**/**H**) were measured using SQUID magnetometers (Quantum Design, MPMS-XL and 1T) between 2 and 400 K in different applied magnetic fields under both zero-field-cooled (ZFC) and field-cooled (FC) conditions. FC measurements were performed on cooling (FCC) from high temperatures to 2 K after the ZFC measurements. In all ZFC measurements, samples were rapidly (within 3–5 min) inserted into a magnetometer, which was kept at 10 K; then, temperature was set to 2 K, and finally a measurement magnetic field was applied. Isothermal magnetization measurements (M versus H) were performed between −70 and 70 kOe at 2 K and 300 K. Specific heat, *C*_p_, was recorded between 2 and 300 K on cooling at 0 and 90 kOe by a pulse relaxation method using a commercial calorimeter (Quantum Design PPMS). ^57^Fe Mössbauer spectra were recorded at 300 K using a conventional constant-acceleration spectrometer MS-1104Em in the transmission geometry. The radiation source ^57^Co(Rh) was kept at RT. All isomer shifts are referred to *α*-Fe at 300 K. The experimental spectra were processed and analysed using methods of spectral simulations implemented in the SpectrRelax program [19]. Differential scanning calorimetry (DSC) curves of (Sc_0.95_Co_0.05_)CoO_3_ powder were recorded on a Mettler Toledo DSC1 STAR^e^ system at a heating/cooling rate of 10 K min^−1^ between 290 K and 873 K in open Al capsules; no DSC anomalies were detected, and the sample remained single-phase after the DSC experiment.

## 3. Results and discussion

**Figure 1.**
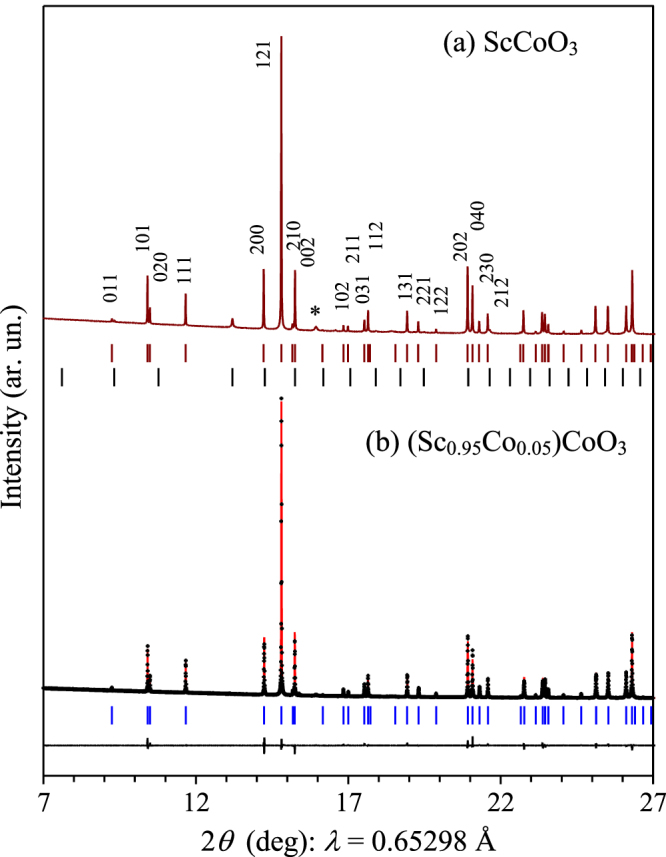
(a) A portion of the experimental synchrotron XRPD pattern of ScCoO_3_. The bars show possible Bragg reflection positions for the perovskite phase and Sc_2_O_3_ impurity (from top to bottom); *hkl* indexes for some reflections are given. A star marks a reflection from Au (a contamination from an Au capsule). (b) Portions of experimental (black crosses), calculated (red line) and difference (black line) synchrotron XRPD patterns for (Sc_0.95_Co_0.05_)CoO_3_. The bars show possible Bragg reflection positions for the perovskite phase.

The stoichiometric ScCoO_3_ sample contained Sc_2_O_3_ impurity (figure 1(a)) suggesting that the composition of the main perovskite phase is shifted according to the scheme:1
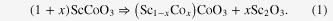



The amount of Sc_2_O_3_ was estimated to be 3–5 weight % from the Rietveld fitting of different laboratory XRPD data. The Rietveld refinement of the synchrotron XRPD data (figure 1(a)) gave about 7.7 weight % of Sc_2_O_3_. A sample with the total chemical composition of Sc_0.9_CoO_2.85_ (≈(Sc_0.95_Co_0.05_)CoO_3_) was prepared without impurities. The EPMA showed that the Sc:Co ratio was 0.901(11):1 in Sc_0.9_CoO_2.85_, in very good agreement with the target chemical composition. No other chemical elements (such as, K and Cl) were detected in Sc_0.9_CoO_2.85_.

The structural analysis showed that all cation and oxygen sites are fully occupied in Sc_0.9_CoO_2.85_ suggesting the (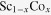)CoO_3_ composition with *x* ≈ 0.0526. Structural parameters of (Sc_0.95_Co_0.05_)CoO_3_ are summarized in table 1, and selected bond lengths, angles and bond-valence sums (BVS) [20] in table 2. The BVS values of all the cation sites are close to the formal ionic values of +3. Experimental, calculated and difference synchrotron XRPD profiles are shown in figure 1(b). (Sc_0.95_Co_0.05_)CoO_3_ crystallizes in the GdFeO_3_-type structure with space group *Pnma* similar to other members of the perovskite cobaltite family *A*CoO_3_ (*A*^3+^ = Y and Pr-Lu) (except for LaCoO_3_) [6, 12]. (Sc_0.95_Co_0.05_)CoO_3_ has just two cation positions: the first position is for the *A* cation, and the second for the *B* cation. The lattice parameters and unit cell volume of (Sc_0.95_Co_0.05_)CoO_3_ follow the general trends observed in the *A*CoO_3_ (*A*^3+^ = Y and Pr-Lu) family with low-spin Co^3+^ ions [12]. However, the unit cell volume of (Sc_0.95_Co_0.05_)CoO_3_ (*V* = 185.141 Å^3^) with the Co^3+^ radius of 0.545 Å [21] is smaller than that of ScAlO_3_ (*V* = 185.915 Å^3^) with the Al^3+^ radius of 0.535 Å probably because of the shift from the stoichiometric composition [12].

**Table 1.** Structure parameters of (Sc_0.95_Co_0.05_)CoO_3_ and (Sc_0.95_*M*_0.05_)*M*O_3_ (*M* = Co_0.75_Cr_0.25_) at room temperature.

Site	Wyckoff position	*X*	*y*	*z*	*B* (Å^2^)
(Sc_0.95_Co_0.05_)CoO_3_					
Sc/Co	4*c*	0.07704(8)	0.25	0.97603(12)	0.333(9)
Co	4*b*	0	0	0.5	0.158(7)
O1	4*c*	0.4468(3)	0.25	0.1313(3)	0.16(3)
O2	8*d*	0.3101(2)	0.0631(2)	0.6830(2)	0.22(2)
(Sc_0.95_*M*_0.05_)*M*O_3_ (*M* = Co_0.75_Cr_0.25_)					
Sc/*M*	4*c*	0.07621(8)	0.25	0.97673(14)	0.511(11)
*M*	4*b*	0	0	0.5	0.311(8)
O1	4*c*	0.4465(3)	0.25	0.1307(3)	0.20(4)
O2	8*d*	0.3101(3)	0.06395(18)	0.6838(3)	0.35(3)

The occupation factor of all sites is unity. Statistical distribution according to the compositions was assumed for the Sc/Co, Sc/*M* and *M* sites.

Space group *Pnma* (No 62); *Z* = 4.

(Sc_0.95_Co_0.05_)CoO_3_: *a* = 5.26766(1) Å, *b* = 7.14027(2) Å, *c* = 4.92231(1) Å, and *V* = 185.141(1) Å^3^; *R*_wp_ = 3.72%, *R*_p_ = 2.43%, *R*_B_ = 2.08%, and *R*_F_ = 1.44%; *ρ*_cal_ = 5.474 g cm^−3^.

(Sc_0.95_*M*_0.05_)*M*O_3_: *a* = 5.29435(1) Å, *b* = 7.20430(2) Å, *c* = 4.95170(1) Å, and *V* = 188.868(1) Å^3^; *R*_wp_ = 3.04%, *R*_p_ = 2.17%, *R*_B_ = 3.83%, and *R*_F_ = 2.85%; *ρ*_cal_ = 5.296 g cm^−3^.

**Table 2.** Selected bond lengths, *l* (Å) < 3.0 Å, bond angles (deg) and bond valence sums (BVS) in (Sc_0.95_Co_0.05_)CoO_3_ and (Sc_0.95_*M*_0.05_)*M*O_3_ (*M* = Co_0.75_Cr_0.25_)^a^.

(Sc_0.95_Co_0.05_)CoO_3_		(Sc_0.95_*M*_0.05_)*M*O_3_ (*M* = Co_0.75_Cr_0.25_)	
Sc—O1	2.051(2)	Sc—O1	2.061(2)
Sc—O2	2.091(1) × 2	Sc—O2	2.101(1) × 2
Sc—O1	2.093(2)	Sc—O1	2.104(2)
Sc—O2	2.317(1) × 2	Sc—O2	2.331(1) × 2
Sc—O2	2.528(1) × 2	Sc—O2	2.555(1) × 2
BVS(Sc^3+^)	3.02	BVS(Sc^3+^)	2.92
Co—O2	1.908(1) × 2	*M*—O2	1.917(1) × 2
Co—O2	1.919(1) × 2	*M*—O2	1.933(1) × 2
Co—O1	1.919(1) × 2	*M*—O1	1.935(1) × 2
BVS(Co^3+^)	3.35	BVS(Co^3+^)	3.24
Co—O1—Co	136.93(5)	*M*—O1—*M*	137.16(5)
Co—O2—Co	140.79(5) × 2	*M*—O2—*M*	140.61(5) × 2

a BVS = 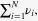 *ν*_*I*_ = exp[(*R*_0_—*l*_*i*_)/*B*], *N* is the coordination number, *B* = 0.37, *R*_0_(Sc^3+^) = 1.849, and *R*_0_(Co^3+^) = 1.70 [20].

**Figure 2.**
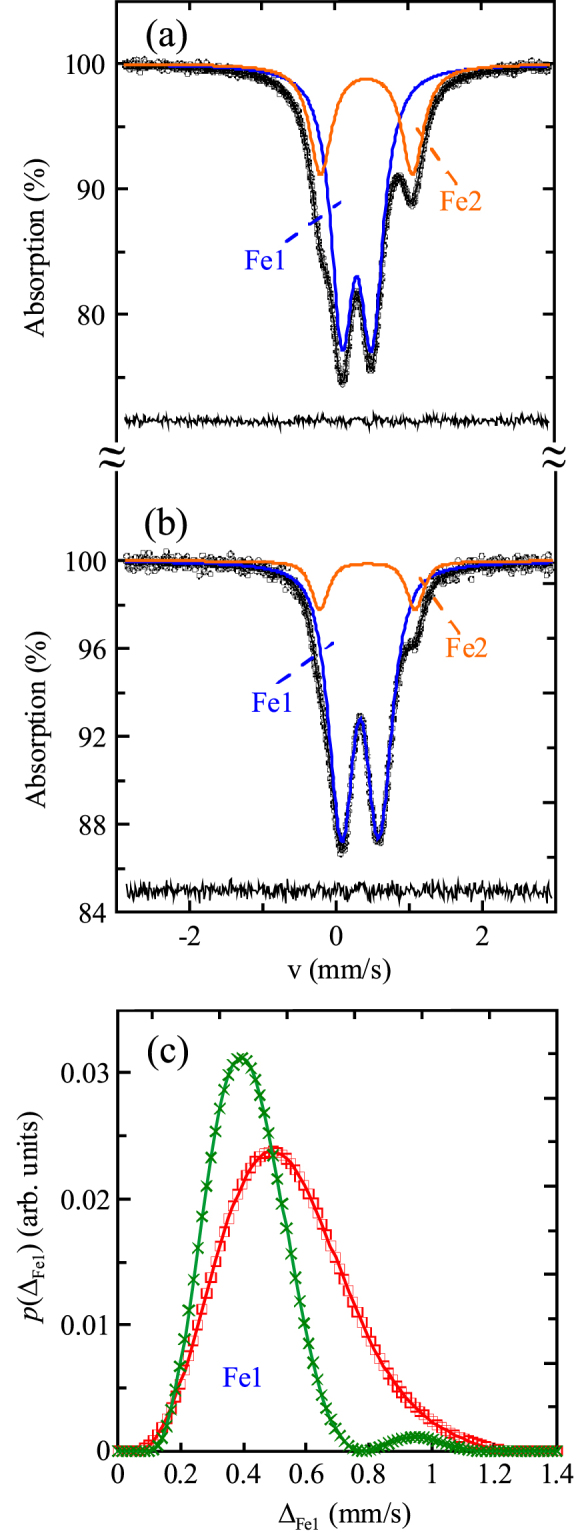
^57^Fe Mössbauer spectra at 300 K and fitting results for (a) (Sc_0.95_*M*_0.05_)*M*O_3_ (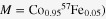 and (b) (Sc_0.89_*M*_0.11_)*M*O_3_ (*M* = Co_0.6_Fe_0.4_). The difference between the experimental and calculated spectra is shown at the bottom. (c) Distribution functions *p*(*Δ*_Fe1_) of the quadrupole splitting *Δ*_Fe1_ for the Fe1 subspectrum in (Sc_0.95_*M*_0.05_)*M*O_3_ (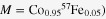 (green crosses) and (Sc_0.89_*M*_0.11_)*M*O_3_ (*M* = Co_0.6_Fe_0.4_) (red squares).

The Mössbauer spectrum of (Sc_0.95_*M*_0.05_)*M*O_3_ (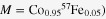 at 300 K is shown on figure 2(a). It clearly consists of two doublets, Fe1 and Fe2, whose isomer shift (*δ*_Fe1_ < *δ*_Fe2_) and quadrupole splitting (*Δ*_Fe1_ < *Δ*_Fe2_) values indicate that the high-spin (HS) Fe^3+^ ions occupy two positions with different oxygen surrounding. The existence of these doublets could only originate from ^57^Fe^3+^ ions in positions corresponding to the *A* and *B* sublattices. The *δ*_Fe1_ (=0.32(1) mm s^−1^) and *Δ*_Fe1_ (=0.42(1) mm s^−1^) values for the first Fe1 doublet are in good agreement with the *δ* = 0.31–0.33 mm s^−1^ and *Δ* = 0.38–0.50 mm s^−1^ values for Fe^3+^ ions located in the *B* site of 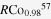
Fe_0.02_O_3_ (*R* = Y, Eu and Lu) perovskites [22]. Taking into account that an increase in the average 〈Fe-O〉 distances leads generally to an increase in *δ* values [23], the Fe2 doublet with the larger isomer shift of *δ*_Fe2_ = 0.45(1) mm s^−1^ should correspond to ^57^Fe^3+^ ions located at the larger *A* site, and the larger quadrupole splitting of *Δ*_Fe2_ = 1.26(1) mm s^−1^ indicates that Fe^3+^ ions have highly asymmetric coordination at the *A* site. It is expected that smaller 3*d* transition metals (Cr^3+^, Fe^3+^ and Co^3+^) should be displaced off the position occupied by the larger Sc^3+^ ions similar to the displacement of Mn^2+^ ions found in Sr_0.98_Mn_0.02_TiO_3_ [10]. Based on experimental values of the areas of the two doublets (*I*_Fe_ values in table 3), the distribution of ^57^Fe^3+^ ions is not statistical (the statistical distribution would result in about 5% of ^57^Fe^3+^ ions at the *A* site), but ^57^Fe^3+^ ions preferably occupy the *A* site (about 30%).

**Table 3.** Hyperfine parameters of the ^57^Fe Mössbauer spectra of (Sc_0.95_*M*_0.05_)*M*O_3_ (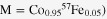 and (Sc_0.89_*M*_0.11_)*M*O_3_ (*M* = Co_0.6_Fe_0.4_) at 300 K.

Sample	Sites	*δ* (mm s^−1^)	*Δ* (mm s^−1^)	*W* (mm s^−1^)	*I* (%)
(Sc_0.95_*M*_0.05_)*M*O_3_	Fe1	0.32(1)^a^	0.42(1)^a^	0.31(2)	69(1)
(*M* = Co_0.95_Fe_0.05_)	Fe2	0.45(2)	1.26(2)	0.34(1)	31(2)
(Sc_0.89_*M*_0.11_)*M*O_3_	Fe1	0.33(2)^a^	0.55(3)^a^	0.25(2)	88(2)
(*M* = Co_0.6_Fe_0.4_)	Fe2	0.43(2)	1.30(2)	0.28(1)	12(2)

a These are the average 〈*δ*_Fe1_〉 and 〈*Δ*_Fe1_〉 values obtained from the distribution functions *p*(*δ*_Fe1_) and *p*(*Δ*_Fe1_).

*δ* is an isomer shift, *Δ* is quadrupole splitting, *W* is linewidth, and *I* is a relative intensity.

To verify the correctness of the doublet assignment to the *A* and *B* positions, we calculated a lattice contribution (***V***^lat^) to the electric field gradient (EFG) tensor at ^57^Fe at the *A* and *B* positions, using the experimental crystallographic data of (Sc_0.95_Co_0.05_)CoO_3_ (table 1). After diagonalization, the main EFG tensor components (|*V*_ZZ_| ≥ |*V*_XX_| ≥ |*V*_YY_|) were used to estimate the theoretical quadrupole splitting *Δ*^theor^ = *eQV*_ZZ_/2(1 + *η*^2^/3)^1/2^, where *η* ≡ (*V*_XX_–*V*_YY_)/*V*_ZZ_ is the parameter of asymmetry of EFG. The best agreement between the theoretical (*Δ*_*B*_^theor^ = 0.32 mm s^−1^ and *Δ*_*A*_^theor^ = 0.56 mm s^−1^) and experimental values (table 3) of quadrupole splitting (*Δ*_i_^exp^) was obtained for the oxygen dipole polarizability of *α*_О_ ≈ 0.6 Å^3^ (for nominal charges of *Z*_O_ = −2, *Z*_Sc_ = +3 and *Z*_Co_ = +3, and the quadrupole moment of ^57^Fe nuclei of *Q* = 0.21 barns [24]). The obtained high value of *α*_О_ agrees well with the data for other oxides [25]. The main factors, which can be responsible for the observed discrepancy between the *Δ*_i_^theor^ and *Δ*_i_^exp^ values, are the uncertainty in choosing the effective charges on the ions (Sc, Co and O) and the nucleus quadrupole moment *eQ* for ^57^Fe nuclei [24]. However, our calculations qualitatively correctly predict the ratio of the *Δ*_i_ values (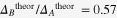 and 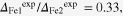 see table 3) thus confirming that our model, which was used for the fitting and interpretation of the experimental spectrum, is reliable, and the Fe1 and Fe2 doublets are correctly assigned to the *B* and *A* positions, respectively, in the structure of (Sc_0.95_*M*_0.05_)*M*O_3_ (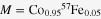).

**Figure 3.**
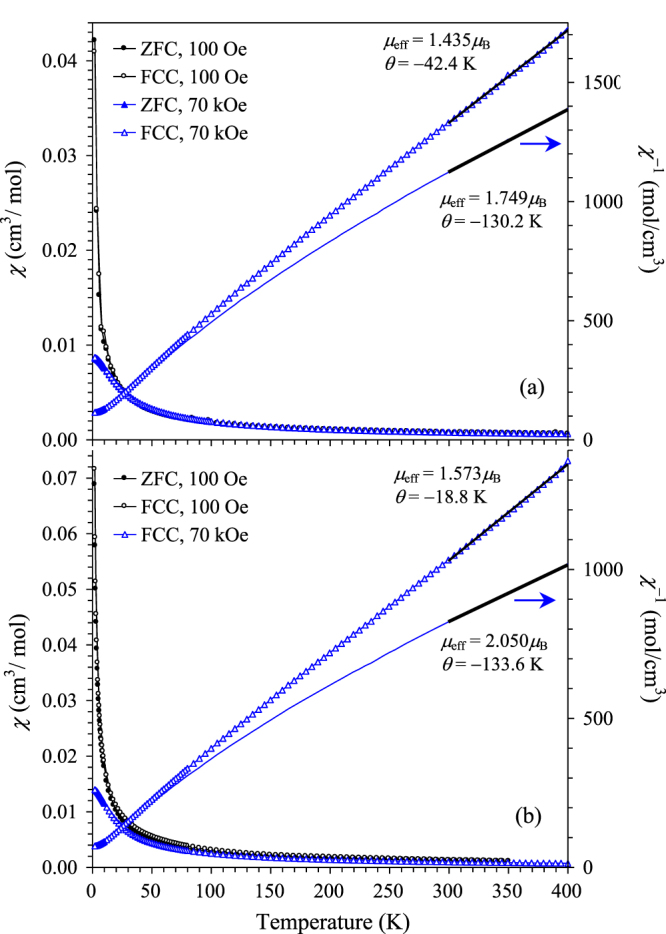
ZFC (filled symbols) and FCC (empty symbols) uncorrected magnetic susceptibility (*χ* = M/H) curves at 100 Oe and 70 kOe for (a) (Sc_0.95_Co_0.05_)CoO_3_ and (b) (Sc_0.95_*M*_0.05_)*M*O_3_ (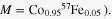 The right-hand axes give inverse FCC curves (*χ*^−1^ versus *T*) at 70 kOe. Parameters (*μ*_eff_ and *θ*) of the Curie–Weiss fits (bold lines) between 300 and 400 K are given. The thin lines show the same FCC *χ*^−1^ versus *T* curves at 70 kOe corrected for contributions from diamagnetic sample holders and core diamagnetism.

**Figure 4.**
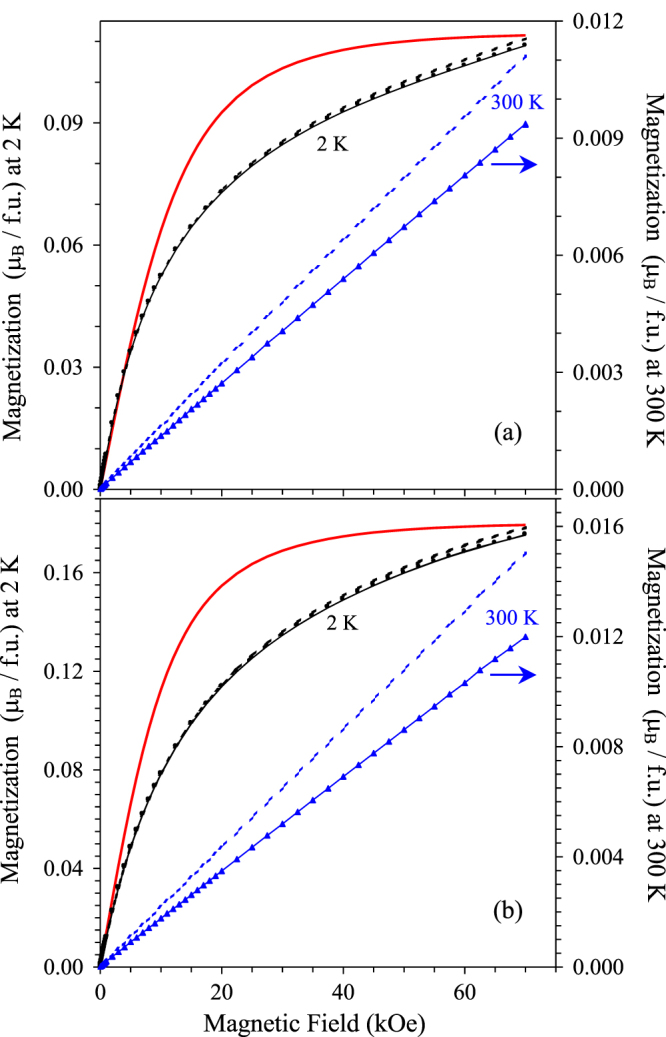
(a) Uncorrected M versus H curves of (Sc_0.95_Co_0.05_)CoO_3_ at 2 K and 300 K (symbols with the line). The line shows the Brillouin function with *g* = 2 and *S* = 2 at 2 K, multiplied by 0.028. (b) M versus H curves of (Sc_0.95_*M*_0.05_)*M*O_3_ (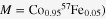 at 2 K and 300 K (symbols with the line). The line shows the Brillouin function with *g* = 2 and *S* = 5/2 at 2 K, multiplied by 0.036. Broken lines show M versus H curves corrected for diamagnetic sample holders and core diamagnetism.

We observed no difference between the ZFC and FCC curves measured at low magnetic fields (e.g., 0.1 kOe) and high magnetic fields (e.g., 70 kOe) (figure 3(a)). At high temperatures, almost no difference was found in magnetic susceptibilities measured at 0.1 and 70 kOe; however, at low temperatures, magnetic susceptibilities were suppressed by high magnetic fields in agreement with the isothermal M versus H curves (figure 4(a)). (Sc_0.95_Co_0.05_)CoO_3_ exhibits paramagnetic behaviour (figure 3(a)) with a relatively large effective magnetic moment of *μ*_eff_ = 1.749(6)*μ*_B_/f.u. (*μ*_B_ is the Bohr magneton and f.u. is the formula unit) and the Curie–Weiss temperature of *θ* = −130(3) K. It is expected that Co^3+^ ions at the *B* site should be in the non-magnetic low-spin (LS) state similar to other members of the *A*CoO_3_ (*A*^3+^ = Y and Pr-Lu) family [6, 26]; the temperature of the spin-state (LS-to-HS) transition increases sharply with decreasing the size of the *A* type cation [6]. Therefore, a large effective magnetic moment should originate from the high-spin Co^3+^ ions located at the *A* site. The expected calculated effective magnetic moment is 1.124*μ*_B_ (for 0.0526Co^3+^), which is close to the experimentally obtained value. Large effective magnetic moments and Curie–Weiss temperatures were also observed in LaCo_1−x_*M*_x_O_3_ (*M* = Rh and Ir) [27]; *μ*_eff_ for the impurity-related magnetism is usually one order of magnitude smaller [28]. Magnetic properties of (Sc_0.95_*M*_0.05_)*M*O_3_ (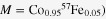 were very similar with those of (Sc_0.95_Co_0.05_)CoO_3_ (figure 3(b)), with a slightly larger *μ*_eff_ = 2.050(4)*μ*_B_/f.u. because of the presence of Fe^3+^ ions (the expected *μ*_eff_ is about 1.63*μ*_B_). Note that the intrinsic magnetic moment of (Sc_0.95_Co_0.05_)CoO_3_ is quite small at high temperatures; therefore, diamagnetic contributions (from sample holders and core diamagnetism) have a significant influence on the *μ*_eff_ and *θ* values (figure 3) making it difficult to discuss them.

**Figure 5.**
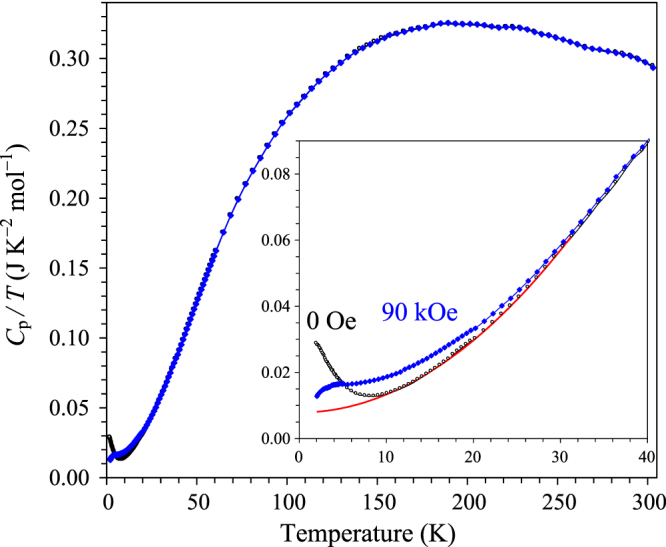
Specific heat data of (Sc_0.95_Co_0.05_)CoO_3_ (powder was washed from KCl and then pressed into pellets at 3 GPa) at a zero magnetic field (white circles) and 90 kOe (blue diamonds) plotted as *C*_p_/*T* versus *T*. The inset shows the details below 40 K; the red line is the fit with the equation *C*_p_/*T* = *γ* + *β*_1_*T*^2^ between 9 and 31 K.

The isothermal *M* versus *H* curves of (Sc_0.95_Co_0.05_)CoO_3_ and (Sc_0.95_*M*_0.05_)*M*O_3_ (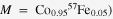 showed no hysteresis and passed through the origin (figure 4); no saturation behaviour was also observed at 2 K, in contrast with the expected property for free ions, that is, the Brillouin function behaviour. The *M* versus *H* curve of (Sc_0.95_Co_0.05_)CoO_3_ was linear at 300 K up to 70 kOe. Deviations from the Brillouin function behaviour was observed in some doped LaCoO_3_ samples [29]. Specific heat of (Sc_0.95_Co_0.05_)CoO_3_ is given on figure 5; between 9 and 31 K, the data follow the equation *C*_p_/*T* = *γ* + *β*_1_*T*^2^ with *γ* = 7.86(8) mJmol^−1^ K^−2^ and *β*_1_ = 0.05452(17) mJmol^−1^ K^−4^ (the line in the inset of figure 5). Taking into account the fact that (Sc_0.95_Co_0.05_)CoO_3_ is an insulator, the upturn of the *C*_p_/*T* values below 9 K and the apparent electronic contribution *γ* could originate from Schottky-type contributions or single-ion excitations. The *β*_1_ value of (Sc_0.95_Co_0.05_)CoO_3_ was close to that of ScRhO_3_ (*β*_1_ = 0.0589 mJmol^−1^ K^−4^) [28].

**Figure 6.**
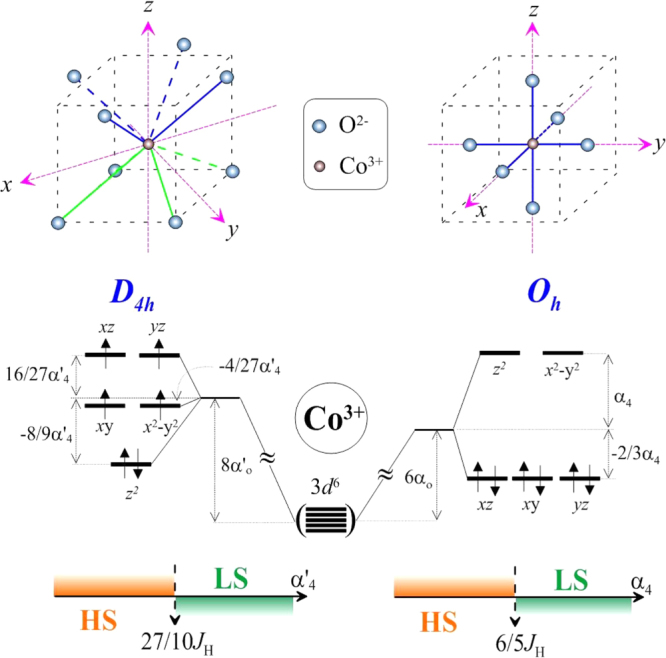
Schematic presentations of the energy diagrams for the Co^3+^ ion in the idealized *A* site with the *D*_4*h*_ symmetry and in the idealized *B* site with the *O*_*h*_ symmetry in (Sc_0.95_Co_0.05_)CoO_3_ (where *α*_0_ and *α*_4_ are radial integrals, *J*_H_ is the intra-atomic Hund energy).

The presence of Co^3+^ ions in the LS and HS states is in qualitative agreement with the energy diagrams of Co^3+^ in crystal fields with local symmetries of *O*_*h*_ (for the *B* position, in the first approximation) and *D*_4*h*_ (for the *A* position, in the first approximation) (figure 6) [30]. In the case of the same average bond distances 〈Co-O〉, the crystal field splitting, 5/3*α*_4_ (where *α*_4_ ∼ 1/[〈Co-O〉]^5^ is a radial integral), of Co^3+^ orbitals for the *O*_*h*_ octahedral site is higher than the crystal field splitting for the *D*_4*h*_ site (16/27*α*′_4_). Moreover, the average 〈Co-O〉 bond distances are longer in the *A* position in comparison with the *B* position (table 2), thus, further reducing the 16/27*α*′_4_ value and the crystal field splitting. In the case of the *O*_*h*_ octahedral site, where the LS state of Co^3+^ is experimentally realized, it gives *α*_4_ > 6/5*J*_H_, where *J*_H_ is the intraatomic Hund energy, [E_LS_–E_HS_ = (6×(−2/3*α*_4_) + 2 × (−3*J*_H_))–(1×(−2/3*α*_4_) + (−10*J*_H_)) < 0]. In the case of the *D*_4*h*_ site, the LS state of Co^3+^ will only be realized at *α*′_4_ > 27/10*J*_H_, [E_LS_–E_HS_ = (2×(−8/9*α*′_4_) + 4×(−4/27*α*′_4_) + 2×(−3*J*_H_)) –(1×(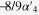) + (−10*J*_H_)) < 0]. Considering that *α*′_4_ should be smaller than *α*_4_ (and with the same *J*_H_ for Co^3+^), the above conditions result in the HS state of Co^3+^ at the *D*_4*h*_ site. Note that the HS state of Co^3+^ was experimentally found in BiCoO_3_ [31], where Co^3+^ ions are located in a square pyramidal coordination.

**Figure 7.**
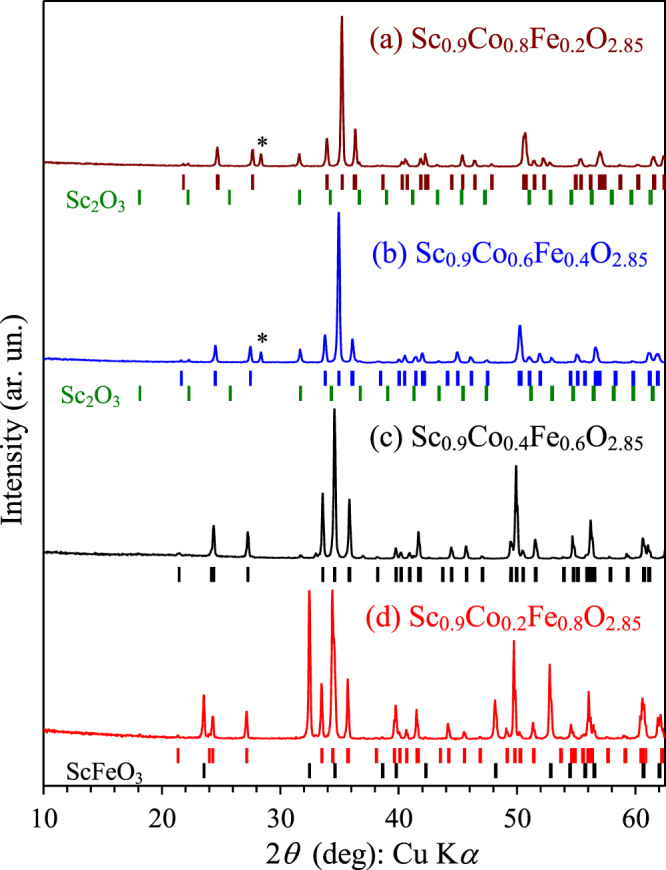
Portions of XRPD patterns of samples with the total composition of Sc_0.9_Co_1−*x*_Fe_*x*_O_2.85_ with *x* = 0.2, 0.4, 0.6 and 0.8. The bars show possible Bragg reflection positions for the perovskite phase and Sc_2_O_3_ impurity (from top to bottom) on *a*–*c*. A star marks a reflection from KCl in unwashed samples. On *d*, the bars show possible Bragg reflection positions for the perovskite phase and ScFeO_3_ impurity (from top to bottom).

**Figure 8.**
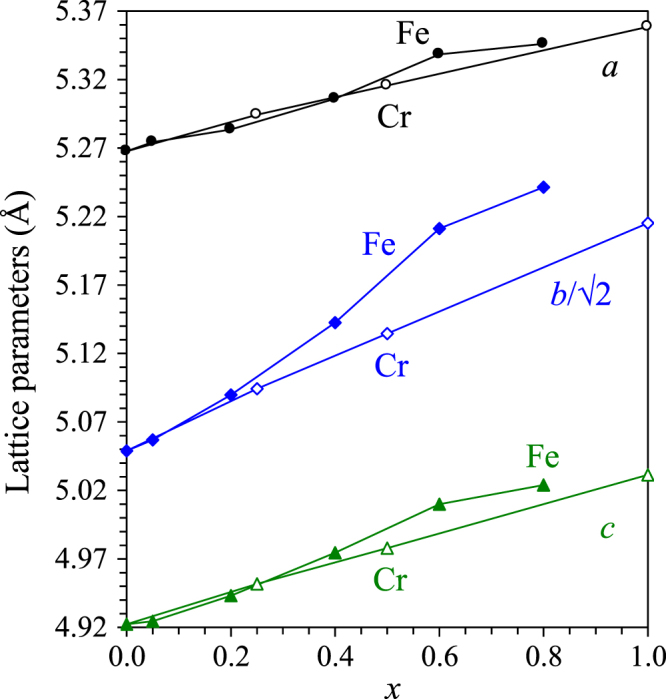
Lattice parameters versus the composition for Sc_0.9_Co_1−*x*_ Cr_*x*_O_2.85_ (*x* = 0, 0.25 and 0.5) and ScCrO_3_ [36] (empty symbols) and Sc_0.9_Co_1−*x*_Fe_*x*_O_2.85_ (*x* = 0, 0.05, 0.2, 0.4, 0.6 and 0.8) (filled symbols).

By the analogy with Sc_0.9_CoO_2.85_, we prepared solid solutions with the total composition of Sc_0.9_Co_1−*x*_Fe_*x*_O_2.85_ (*x* = 0.2, 0.4, 0.6 and 0.8). However, the samples with *x* = 0.2, 0.4 and 0.6 contained Sc_2_O_3_ impurity (figure 7) suggesting that the chemical composition of the perovskite phases is further shifted. Sc_0.9_Co_0.2_Fe_0.8_O_2.85_ already contained a large amount of ScFeO_3_ impurity with the corundum structure [32]. The lattice parameters of the solid solutions 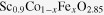 are shown on figure 8. Monotonic changes of the lattice parameters were found with a deviation for the two-phase sample with *x* = 0.8; this fact suggests that the solid solution limit is near *x* = 0.7.

Almost single-phase Sc_0.8_Co_0.6_Fe_0.4_O_2.7_ (≈(Sc_0.89_*M*_0.11_)*M*O_3_ with *M* = Co_0.6_Fe_0.4_) was prepared whose Mössbauer spectrum at 300 K is shown on figure 2(b). The spectrum also consisted of two quadrupole doublets, Fe1 and Fe2, but in contrast to (Sc_0.95_*M*_0.05_)*M*O_3_ (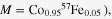 the most intense doublet Fe1 had broadened and asymmetrical components that could be caused by the existence of different configurations {(6-*m*)Co^3+^, *m*Fe^3+^} in the local surrounding of the Fe^3+^ ions within the *B* sublattice. We fitted the experimental spectrum as a superposition of a discrete doublet Fe2 and a distribution *p*(*Δ*_Fe1_) of the quadrupole splittings (*Δ*_Fe1_), assuming a linear relation between *Δ*_Fe1_ and *δ*_Fe1_ [33]. For comparison, a similar fitting analysis was carried out for (Sc_0.95_*M*_0.05_)*M*O_3_ (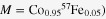 (table 3); note that the Mössbauer parameters for this sample in two models (the first model is two discrete doublets, and the second one with a distribution for Fe1) were almost identical. The obtained *p*(*Δ*_Fe1_) distributions are shown in figure 2(c), and the best-fit hyperfine parameters (average 〈*δ*_Fe1_〉 and 〈*Δ*_Fe1_〉 values for the Fe1 subspectra) and relative intensities (*I*_i_) of the partial spectra are listed in table 3. A comparison of these data shows that changing the iron content in the samples does not significantly affect hyperfine parameters of the Fe1 and Fe2 doublets, while their relative intensities undergo some changes. According to the experimental intensity ratio of the partial spectra, *I*_Fe1_/*I*_Fe2_ (table 3), in the case of (Sc_0.89_*M*_0.11_)*M*O_3_ (*M* = Co_0.6_Fe_0.4_), Fe^3+^ ions were distributed almost statistically between the *A* and *B* sites (statistical distribution would give 10% of Fe^3+^ at the *A* site, and the experimental doublet area is 12(2)%). The resulting distribution *p*(*Δ*_Fe1_) for (Sc_0.95_*M*_0.05_)*M*O_3_ (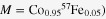 is narrow and has symmetrical profile, thus, indicating a uniform nearest surrounding of Fe^3+^ ions.

The location of small Fe^3+^ ions at the *A* site of classical *AB*O_3_ perovskites is quite unusual, especially their strong preference to occupy the *A* site at small doping levels. To the best of our knowledge, (Sc_1−*x*_*M*_*x*_)*M*O_3_ compounds are the first example of such behaviour. It should be noted that Fe^3+^ ions were found by the Mössbauer spectroscopy at the *A*′ site of *A*-site ordered perovskites with the general composition of *AA*′_3_*B*_4_O_12_, for example, in CaCu_3_Fe_4_O_12_ [34] and CaMn_3_Mn_4_O_12_ [35]. However, Fe^3+^ ions substitute for Cu^2+^ or Mn^3+^ ions—other transition metals—in a special *A*′ position, whose coordination environment (square-coordinated *A*′O_4_) is quite different from a typical coordination of the *A* site in perovskites (*A*O_8_-*A*O_12_).

**Figure 9.**
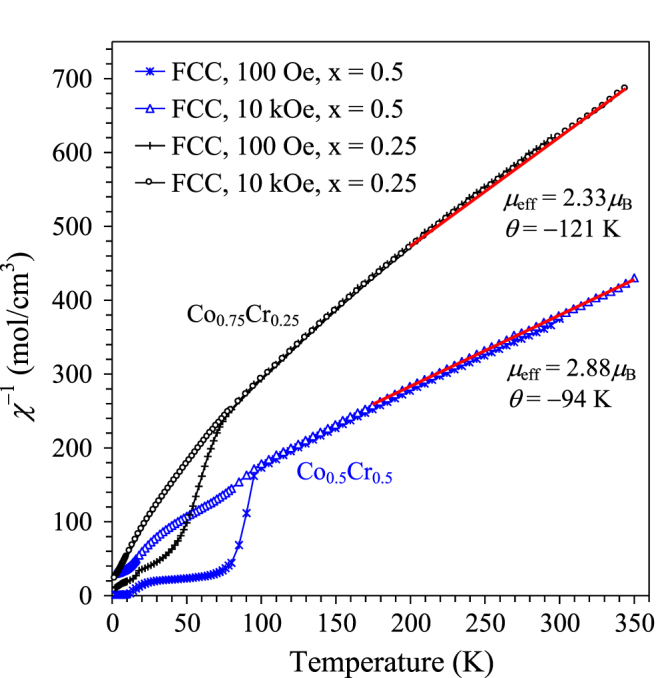
Uncorrected inverse FCC magnetic susceptibility curves (*χ*^−1^ versus *T*) of (Sc_0.95_*M*_0.05_)*M*O_3_ (*M* = Co_1−*x*_Cr_*x*_ with *x* = 0.25 and 0.5) at 100 Oe and 10 kOe. Parameters (*μ*_eff_ and *θ*) of the Curie–Weiss fits (lines) are given.

We also prepared solid solutions with the total composition of Sc_0.9_Co_1−*x*_Cr_*x*_O_2.85_ (*x* = 0.25 and 0.5). The structural parameters of (Sc_0.95_*M*_0.05_)*M*O_3_ (*M* = Co_0.75_Cr_0.25_) are given in tables 1 and 2; and the compositional dependence of the lattice parameters is shown on figure 8. Almost linear changes of the lattice parameters suggest that the solid solutions are formed in the whole compositional range. Inverse magnetic susceptibilities of (Sc_0.95_*M*_0.05_)*M*O_3_ (*M* = Co_0.75_Cr_0.25_ and Co_0.5_Cr_0.5_) are given on figure 9. The *χ*^−1^ values were almost field-independent above 70 K for (Sc_0.95_*M*_0.05_)*M*O_3_ (*M* = Co_0.75_Cr_0.25_) and above 100 K for (Sc_0.95_*M*_0.05_)*M*O_3_ (*M* = Co_0.5_Cr_0.5_) suggesting an impurity-free paramagnetic behaviour. The Curie–Weiss fits of the data corrected for diamagnetic contributions gave *μ*_eff_ = 2.47*μ*_B_/f.u. and *θ* = −144 K for (Sc_0.95_*M*_0.05_)*M*O_3_ (*M* = Co_0.75_Cr_0.25_) with the expected *μ*_eff_ = 2.20*μ*_B_/f.u and *μ*_eff_ = 2.99*μ*_B_/f.u. and *θ* = −107 K for (Sc_0.95_*M*_0.05_)*B*O_3_ (*M* = Co_0.5_Cr_0.5_) with the expected *μ*_eff_ = 2.91*μ*_B_/f.u. The anomalies at 100 Oe below 70 K in (Sc_0.95_*M*_0.05_)*M*O_3_ (*M* = Co_0.75_Cr_0.25_) and below 100 K in (Sc_0.95_*M*_0.05_)*M*O_3_ (*M* = Co_0.5_Cr_0.5_) could originate from the onset of short-range or long-range magnetic interactions.

## 4. Conclusions

We found that ‘ScCoO_3_’-based perovskites are formed as non-stoichiometric (Sc_1−*x*_*M*_*x*_)*M*O_3_ with *x* = 0.05–0.11 and *M* = Co, (Co, Fe) and (Co, Cr) under high pressure (6 GPa) and high temperature (1570 K) conditions. There is evidence that (Sc_0.95_Co_0.05_)CoO_3_ has non-magnetic low-spin Co^3+^ ions at the *B* site and paramagnetic high-spin Co^3+^ ions at the *A* site. In the iron-doped samples (Sc_1−*x*_*M*_*x*_)*M*O_3_ with *M* = (Co, Fe), Fe^3+^ ions have strong preference to occupy the *A* site of such perovskites that is quite unusual for perovskites.
